# Changes in HDL cholesterol, particles, and function associate with pediatric COVID-19 severity

**DOI:** 10.3389/fcvm.2022.1033660

**Published:** 2022-10-12

**Authors:** Michele Mietus-Snyder, William Suslovic, Meghan Delaney, Martin P. Playford, Rami A. Ballout, John R. Barber, James D. Otvos, Roberta L. DeBiasi, Nehal N. Mehta, Alan T. Remaley

**Affiliations:** ^1^Children's National Hospital, Washington, DC, United States; ^2^The Children's National Clinical and Translational Science Institute, Washington, DC, United States; ^3^Division of Cardiology, School of Medicine and Health Sciences, The George Washington University, Washington, DC, United States; ^4^Department of Pediatrics, School of Medicine and Health Sciences, The George Washington University, Washington, DC, United States; ^5^Division of Clinical and Laboratory Medicine, School of Medicine and Health Sciences, The George Washington University, Washington, DC, United States; ^6^Cardiovascular and Pulmonary Branch, National Institutes of Health, Bethesda, MD, United States; ^7^Lipoprotein Metabolism Section, Translational Vascular Medicine Branch, National Heart, Lung, and Blood Institute, National Institutes of Health, Bethesda, MD, United States; ^8^Division of Infectious Diseases, School of Medicine and Health Sciences, The George Washington University, Washington, DC, United States; ^9^Department of Microbiology, Immunology and Tropical Medicine, School of Medicine and Health Sciences, The George Washington University, Washington, DC, United States; ^10^Clinical Center, Department of Laboratory Medicine, National Institutes of Health, Bethesda, MD, United States

**Keywords:** pediatric COVID-19 severity, HDL-cholesterol, HDL subspecies, HDL function, NMR lipoprotein analysis, lipoprotein Z

## Abstract

**Background:**

Myriad roles for high-density lipoprotein (HDL) beyond atheroprotection include immunologic functions implicated in the severity of coronavirus disease-2019 (COVID-19) in adults. We explored whether there is an association between HDL and COVID-19 severity in youth.

**Methods:**

A pediatric cohort (*N* = 102), who tested positive for COVID-19 across a range of disease manifestations from mild or no symptoms, to acute severe symptoms, to the multisystem inflammatory syndrome of children (MIS-C) was identified. Clinical data were collected from the medical record and reserve plasma aliquots were assessed for lipoproteins by NMR spectroscopy and assayed for HDL functional cholesterol efflux capacity (CEC). Findings were compared by COVID-19 status and symptom severity. Lipoprotein, NMR spectroscopy and CEC data were compared with 30 outpatient COVID negative children.

**Results:**

Decreasing HDL cholesterol (HDL-c), apolipoprotein AI (ApoA-I), total, large and small HDL particles and HDL CEC showed a strong and direct linear dose-response relationship with increasing severity of COVID-19 symptoms. Youth with mild or no symptoms closely resembled the uninfected. An atypical lipoprotein that arises in the presence of severe hepatic inflammation, lipoprotein Z (LP-Z), was absent in COVID-19 negative controls but identified more often in youth with the most severe infections and the lowest HDL parameters. The relationship between HDL CEC and symptom severity and ApoA-I remained significant in a multiply adjusted model that also incorporated age, race/ethnicity, the presence of LP-Z and of GlycA, a composite biomarker reflecting multiple acute phase proteins.

**Conclusion:**

HDL parameters, especially HDL function, may help identify youth at risk of more severe consequences of COVID-19 and other novel infectious pathogens.

## Introduction

Patterns of differential susceptibility to the novel severe acute respiratory syndrome coronavirus-2 (SARS-CoV-2) emerged within months of the world's first encounter with coronavirus disease 2019 (COVID-19). Pre-existing conditions identified among the first adult deaths reported in China included advanced age, cardiovascular disease, hypertension, diabetes, respiratory conditions and cancer (1). A subsequent multisite risk stratification of adults admitted for COVID-19 care in the United Kingdom (UK) confirmed these observations and added male sex, obesity, chronic kidney and liver diseases to the list of preexisting conditions associated with metabolic syndrome and higher mortality (2). These findings were later confirmed by analysis of 17 million adult records within the UK National Health System and were extended to include minority race/ethnicity coupled with social deprivation (3).

An early narrative that children were spared from severe COVID-19 outcomes was soon undone. A Kawasaki Disease-like condition was identified following infections in youth in Italy and the UK (4) and soon thereafter in the United States (5) and globally (6). This rare but severe manifestation of COVID-19 has come to be known as the multisystem inflammatory syndrome of children (MIS-C), a post-infectious hyperinflammatory syndrome. Severe acute COVID-19 infections were also increasingly documented across the pediatric age range but with higher prevalence in older adolescents (>15 years) with comorbidities aligning with adult predisposing conditions (7). Disproportionate representation of non-Hispanic Black and Hispanic youth among pediatric COVID-19 cases was also noted (5). The differential burden of both infection (8) and disease severity (9) imposed on minority youth at socioeconomic disadvantage has mirrored the impact of health disparity of this pandemic already described among adults.

One factor common to virtually all pre-existing conditions identified in the adult experience with COVID-19, notably advanced age (10), male sex (11), obesity (12), hypertension (13), diabetes (14), chronic kidney disease (15), metabolic syndrome (16), and health disparity (17, 18) is low levels of high-density lipoprotein cholesterol (HDL-c). Perhaps more importantly, several of these conditions, notably obesity, metabolic syndrome, and diabetes are also associated with diminished HDL function (19, 20). Furthermore, the acute impact of COVID infection on HDL cholesterol, particle subspecies (21) and function have been recognized in adults and associated with disease severity (20).

The purpose of this study was to explore whether an association between HDL function, specifically cholesterol efflux capacity (CEC), HDL-c, HDL particle subspecies and other parameters measured by nuclear magnetic resonance (NMR) lipoprotein profiling correlate with COVID-19 severity in youth.

## Methods

COVID-19, for the purposes of describing study subjects, will be referred to as COVID. All patients included in this study were cared for at Children's National Hospital (CNH), a freestanding quaternary academic medical center serving an urban/suburban population in the Washington, DC metropolitan region. Retrospective analysis of de-identified remnant frozen plasma from hospitalized COVID-positive subjects and COVID-negative outpatient controls was performed with approval from the Children's National Institutional Review Board. Clinical data acquired through chart review by an “honest broker” (WS) designated on the IRB protocol was deidentified for further analysis.

### Subjects

All the SARS-CoV02-positive subjects were sampled before pediatric COVID-19 vaccination was available. Nine patients ≥18 years of age still followed by pediatric subspecialties for preexisting comorbid conditions, were sampled before vaccination became available for adults in the region. COVID-positive cases grouped as “Mild or no symptoms” were hospitalized for other diagnoses and per hospital pandemic policy screened for COVID-19 and identified as positive. Cases grouped “Severe acute symptoms” were hospitalized to manage COVID respiratory and/or gastroenterological symptoms but did not meet criteria for MIS-C. The diagnosis of MIS-C was made based on institutional protocol and Centers for Disease Control and Prevention (CDC) criteria evaluated by the Children's National Hospital MIS-C taskforce, including detectable SARS-CoV-2 antibody or polymerase chain reaction (PCR), and/or an identified laboratory-confirmed COVID positive contact within the previous 4 weeks; probable contact meeting CDC case definition other than laboratory confirmation of SARS-CoV-2 infection or known contact, but with appropriate temporal onset following surge in community SARS-Co-V2 circulation. SARS-CoV-2 antibody was detected in 97% (30/31) of confirmed cases of MIS-C. One case did not have antibody testing performed but had alternate confirmation by PCR testing prior to hospitalization. Because limited pediatric normative data are available for HDL functional assays, “COVID-negative control” (Controls) plasma aliquots were included in the lipoprotein analyses. Controls were sampled at the time of routine outpatient blood draws. No controls had tested positive for SARS-CoV2 in the 30 days prior to enrollment.

### Routine clinical assessment and laboratory analyses

Clinical assessments and laboratory tests included for hospitalized patients were performed on the same day reserve plasma was taken for NMR lipoprotein analyses and HDL functional assays. All laboratory tests included in this study were performed in the CNH clinical laboratory.

### Lipid and lipoprotein analyses

Blood samples for NMR lipoprotein analyses were collected in either Lithium Heparin or EDTA tubes (Becton-Dickinson, Franklin Lakes, NJ, USA) and processed within 2 h of collection, followed by plasma storage at −80^o^C. Samples were thawed on ice prior to performing NMR LipoProfile^®^ analysis at the National Institutes of Health (NIH), using the LP4 deconvolution algorithm on the Vantera^®^ NMR Clinical Analyzer (LabCorp, Burlington, NC, USA) (22). Cholesterol efflux capacity (CEC) was also performed at the NIH on EDTA samples in duplicate, blinded to group assignment, using a validated cell-based *ex vivo* assay as previously described (23).

### Statistical analyses

Descriptive statistics were used to summarize baseline characteristics in the study population, grouped by COVID-19 status and symptom severity. Due to the frequency of nonparametric data distributions, medians and interquartile ranges represent quantitative variables; counts and proportions represent categorical data. Variables were tested for statistical differences among groups using the Kruskal–Wallis test and either the Chi-square or Fisher's Exact test. Differences that attained statistical significance (*p* < 0.05) were then tested using pairwise Wilcoxon-Rank Sum tests or pairwise Chi-Squares or Fisher's exact test to identify the source of differences. Comparisons between groups were adjusted for multiple testing using a Bonferroni correction at *p* < 0.0083 if Control data were available (six tests) and at *p* < 0.0167 if not (three tests).

Linear regression modeling was performed to evaluate predictors of the differences observed in HDL CEC across COVID severity. Among related/collinear lipid predictors of HDL CEC, apolipoprotein A-I (ApoA-I) was the most strongly correlated and thus used to represent these terms. Other predictors were picked based on *a-priori* hypotheses and differences in the demographics. From significant univariate terms, a multivariate model was generated. Age and race/ethnicity were included to account for differences between Controls and COVID-positive groups. All analyses were performed using SAS V9.4 and tests were two-sided. Box plots for HDL-associated variables that showed the greatest change were performed with Rstudio version 1.1.456.

## Results

### Study population

There were 102 COVID-positive and 30 Controls identified for the study. There were no significant differences found in sex, age, or race/ethnicity among COVID-positive youth, but the Controls were younger than those with severe acute COVID (Table 1). Controls were also represented by more children of non-Hispanic Black race/ethnicity than the COVID-positive youth with severe acute symptoms or MIS-C. Pre-existing medical conditions were most likely to exist in COVID-positive youth without MIS-C, particularly those with severe acute symptoms. Asthma, diabetes, sickle cell disease, genetic syndromes, mental health disorders and epilepsy were the most common pre-existing conditions in study subjects. Obesity was a prevalent pre-existing condition that did not differ among groups, though trended higher with increasing disease severity.

**Table 1:** Baseline characteristics for controls and patients with COVID-19, grouped by symptom severity.

**Variable**	**COVID-19 negative controls^1^** ***N* = 30**	**COVID-19 positive**				
		**Mild or no symptoms^2^** ***N* = 38**	**Severe acute symptoms^3^** ***N* = 33**	**MIS-C^4^** ***N* = 31**	***p*-Value**	Multiple comp^*^
**Sex, no. (%)**						
Female	20 (67%)	21 (55%)	21 (55%)	14 (42%)	0.209	All equal
Male	10 (33%)	17 (45%)	17 (45%)	19 (58%)		
**Age, years**,	2.5 (1.0–13.0)	8.0 (2.0–16.0)	14.0 (4.0–17.0)	8.0 (5.0–13.0)	0.007	1 < 3
*Median (IQR)*						
**Race/Ethnicity no. (%)**						
NonHispanic Black	20 (67%)	13 (34%)	12 (36%)	13 (42%)	0.013	1≠ 2 & 3
Hispanic Other Race	4 (13%)	17 (45%)	15 (45%)	13 (42%)		
NonHispanic White	0 (0%)	4 (11%)	1 (3%)	2 (6%)		
NonHispanic Asian	0 (0%)	0 (0%)	0 (0%)	1 (3%)		
NonHispanic unknown	6 (20%)	4 (11%)	5 (15%)	2 (6%)		
**Underlying diagnoses, no. (%)**						
Any preexisting condition	*N*/A	16 (41%)	28 (85%)	1 (3%)	<0.001	4 < 2 < 3

IQR, interquartile range; kg/m^2^, kilograms per meter^2^; MIS-C, multisystem inflammatory syndrome of children; N, number of participants; no., number of a categorical variable; N/A, no data available.

* Multiple comparisons. Bonferroni adjusted p-values for six comparisons < 0.0083.

### Hospital course

Features of the clinical hospital course for COVID-positive groups were extracted from the electronic medical record are described in Tables 2A,B. None of the Controls were hospitalized. Length of hospital stay, medications received, and level of respiratory support differed predictably by disease severity. The number of hospital diagnostic codes and associated classes of medications prescribed reflected the longer and more complicated hospital stays for the youth diagnosed with severe acute symptoms or MIS-C, as compared with those with mild or no symptoms. Those with MIS-C were the most likely be treated with immunosuppressive and antithrombotic medications.

**Table 2A:** Features of the hospital course for patients with COVID-19.

**Variable**	**COVID-19 positive**				
	**Mild or no symptoms^2^ *N* = 38**	**Severe acute symptoms^3^ *N* = 33**	**MIS-C^4^** ***N* = 31**	***p*-Value**	Multiple comp^*^
**Hospital course**						
Total inpatient days median (IQR)	1.6 (0.7–7.2)	8.6 (1.8–35.1)	10.1 (7.5–13.3)	<0.001	2 < 3 & 4
**Body weight for height (or length if <2 years of age), median (IQR)**						
Body mass index (BMI), Kg/m^2^	18 (15–23) *N* = 24	25 (17–36) *N* = 23	19 (17–21) *N* = 20	0.077	All equal
BMI percentile, %ile	63 (9–87)	94 (50–99)	90 (66–98)	0.114	All equal
*N* ≥ 95th <99th %ile	2 (8%)	4 (18%)	3 (15%)	0.609	All equal
*N* ≥ 99th %ile	4 (17%)	7 (32%)	4 (20%)	0.499	All equal
*N* ≤ 1st %ile	6 (25%)	3 (14%)	1 (5%)	0.217	All equal
**Diagnostic codes during hospitalization, no. (%)**						
Pulmonary & upper respiratory	2 (5%)	24 (72%)	21 (68%)	<0.001	2 < 3 & 4
Cardiac/cardiometabolic	4 (11%)	14 (42%)	21 (68%)	<0.001	2 < 3 & 4
Endocrine	5 (13%)	8 (24%)	2 (6%)	0.143	All equal
GI/Hepatic	8 (21%)	8 (24%)	16 (52%)	0.014	2 < 4
Renal	4 (11%)	10 (30%)	10 (32%)	0.055	All equal
Immunosuppression	1 (3%)	6 (18%)	25 (81%)	<0.001	2 & 3 < 4
Hematologic/Thrombotic	3 (8%)	10 (30%)	14 (45%)	0.001	2 < 4
Psychiatric	8 (21%)	9 (27%)	4 (13%)	0.378	All equal
CNS/seizures	8 (21%)	12 (36%)	5 (16%)	0.140	All equal
Pain	21 (55%)	23 (70%)	28 (90%)	0.006	2 < 4
**Medications, no. (%)**						
Immunoglobulin	0 (0%)	4 (12%)	26 (84%)	<0.001	2 & 3 < 4
Antiviral	1 (3%)	10 (30%)	2 (6%)	0.001	2 < 3
Corticosteroids/immunosuppression	4 (11%)	18 (55%)	26 (84%)	<0.001	2 < 3 < 4
Antithrombotic	5 (13%)	22 (67%)	25 (81%)	<0.001	2 < 3 & 4
Analgesic	23 (61%)	27 (82%)	26 (84%)	0.044	All equal
Antiseizure	6 (16%)	18 (55%)	15 (48%)	0.001	2 < 3 & 4
Glucoregulatory	4 (11%)	8 (24%)	1 (3%)	0.045	All equal
Psychotropic	12 (32%)	18 (55%)	18 (58%)	0.052	All equal
**Level of respiratory support, no. (%)**						
Room air	35 (92%)	13 (39%)	7 (23%)	<0.001	2 ≠ 3 & 4
Nasal canula	2 (5%)	5 (15%)	8 (26%)		
BiPAP or CPAP	0	5 (15%)	8 (26%)		
Ventilator	1 (3%)	10 (30%)	8 (26%)		

CNS, central nervous system; BiPAP, bilevel positive airway pressure; CPAP, continuous positive airway pressure; IQR, interquartile range; kg/m^2^, kilograms per meter^2^; MIS-C, multisystem inflammatory syndrome of children; no., number.

* Multiple comparisons; Bonferroni adjusted for three comparisons, significant *p*-value < 0.0167.

**Table 2B:** Clinical laboratory data for patients with COVID-19.

**Variable**	**COVID-19 positive**				
	**Mild or no symptoms^2^** ***N* = 38**	**Severe acute symptoms^3^** ***N* = 33**	**MIS-C^4^** ***N* = 31**	***p*-Value**	Multiple comp^*^
**Blood pressure, median (IQR)**						
Systolic blood pressure, mmHg	104 (98–117) *N* = 33	111 (103–116) *N* = 33	102 (100–111) *N* = 28	0.548	All equal
Diastolic blood pressure, mmHg	64 (60–75) *N* = 33	67 (59–72) *N* = 33	64 (61–71) *N* = 28	0.859	All equal
**Clinical labs**, median (IQR)						
AST, (IU/L)	25 (20–41) *N* = 20	38 (26–60) *N* = 21	31 (25–37) *N* = 29	0.081	All equal
ALT, (IU/L)	26 (21–35) *N* = 21	36 (28–58) *N* = 21	30 (24–41) *N* = 29	0.063	All equal
BUN, mmol/L	3.6 (2.9–5.0) *N* = 29	3.6 (2.5–5.4) *N* = 30	3.6 (2.5–16) *N* = 31	0.949	All equal
Creatinine, μmol/L	32.7 (23.0–59.2) *N* = 29	38.9 (26.5–57.5) *N* = 30	40.7 (29.2–49.5) *N* = 31	0.858	All equal
C reactive protein, mg/L	1.5 (0.8–3.2) *N* = 10	17.7 (7.3–49.4) *N* = 10	27.8 (4.1–92.8) *N* = 28	0.003	2 < 3 & 4
Hemoglobin, g/L	122 (114–133) *N* = 29	117 (99–126) *N* = 26	100 (91–113) *N* = 30	<0.001	2, & 3 > 4
Hematocrit, %	37.0 (34.2–39.4) *N* = 29	35.4 (32.1–38.8) *N* = 26	30.2 (28.0–33.5) *N* = 29	<0.001	2, & 3 > 4
RDW, fL	13.4 (12.5–14.2) *N* = 29	14.6 (13.3–17.7) *N* = 26	14.2 (13.5–15.6) *N* = 29	0.009	2 < 3
WBC, cells/ml	8.0 (6.0–11.7)	8.7 (6.2–10.7)	6.6 (5.0–9.3)	0.406	All equal
Platelets, cells/ml	338 (249–433)	278 (181–362)	436 (254–605)	0.080	All equal

ALT, alanine transaminase; AST, aspartate transaminase; BUN, blood urea nitrogen; IU/L, international units per liter; IQR, interquartile range; mmHg, millimeters of mercury; mg/dl, milligrams per deciliter; MIS-C, multisystem inflammatory syndrome of children; RDW, red cell distribution width; WBC, white blood cell count.

* Multiple comparisons. Bonferroni adjusted for three comparisons, significant *p*-value < 0.0167.

Blood pressure, hepatic (alanine transaminase and aspartate transaminase) and renal (blood urea nitrogen and creatinine) tests did not vary by disease severity. C-reactive protein (CRP) was higher in youth with severe acute symptoms and in those with MIS-C as compared to those with mild or no symptoms. Both hemoglobin and hematocrit were significantly lower in youth with MIS-C than in all other groups. The red cell distribution width (RDW) was higher in COVID-positive youth with severe acute symptoms in comparison with the group with mild or no symptoms. The total white blood cell and platelet counts did not differ among groups.

### Lipids and lipoproteins

Lipid profiles, phenotyping and HDL CEC are described in Table 3. Total cholesterol and low-density lipoprotein (LDL) cholesterol did not differ by COVID diagnosis. Nor were there differences in total and large LDL particles, but medium LDL particle concentrations were greatest in Controls as compared with COVID-positive severe acute symptoms and MIS-C groups. Small LDL particles were highest among COVID-positive youth with severe acute symptoms. Triglycerides and total, large, small and very small triglyceride-rich lipoproteins (TRL), which include both very low density and intermediate density lipoproteins, were higher in youth with MIS-C as compared with Controls and COVID-positive youth with mild or no symptoms. Both LDL and TRL contain one apolipoprotein B (apoB) molecule per particle; ApoB was also higher in youth with MIS-C.

**Table 3:** Lipid profiles, NMR phenotyping and HDL cholesterol efflux capacity.

**Variable**	**COVID-19 negative controls^1^** ***N* = 30**	**COVID-19 positive**				
		**Mild or no symptoms^2^** ***N* = 38**	**Severe acute symptoms^3^** ***N* = 33**	**MIS-C^4^** ***N* = 31**	***p*-Value**	Multiple comp^*^
**NMR lipids, lipoproteins and metabolites**, median (IQR)						
Total Cholesterol, mmol/L	3.5 (3.2–3.8)	3.8 (3.0–4.7)	4.2 (2.9–5.3)	3.5 (2.5−4.3)	0.421	All equal
Total Triglycerides, mmol/L	0.7 (0.6–0.9)	0.7 (0.6–1.3)	1.0 (0.7–1.7)	1.3 (1.1–1.8)	0.001	1 & 2 < 4
LDL-cholesterol, mmol/L	1.9 (1.5–2.2)	1.8 (1.3–2.5)	1.6 (1.3–2.7)	1.4 (0.7–2.3)	0.085	All equal
HDL-cholesterol, mmol/L	1.3 (1.1–1.6)	1.5 (1.1–1.6)	1.4 (0.7–2.3)	0.7 (0.6–1.1)	<0.001	1 & 2 > 3 1, 2 & 3 > 4
Tot TRL-Particles, nmol/L	63.0 (44.3–78.3)	66.3 (38.8–135.1)	83.9 (55.1–189.5)	159.0 (95.6–214.0)	<0.001	1 & 2 < 4
Very Large TRL-Particles, nmol/L	0.1 (0.1–0.2)	0.2 (0.1–0.3)	0.1 (0.1–0.2)	0.1 (0–0.2)	0.052	All equal
Large TRL-Particles, nmol/L	0.1 (0–0.2)	0.1 (0–0.8)	0.6 (0.1–4.2)	1.0 (0.2–4.8)	<0.001	1 < 4
Medium TRL-particles, nmol/L	1.9 (0–13.0)	3.6 (0.9–19.8)	3.6 (0.2–21.5)	5.0 (1.4–13.6)	0.740	All equal
Small TRL-particles, nmol/L	27.9 (17.9–41.7)	25.6 (14.2–41.2)	27.4 (13.5–48.7)	53.8 (33.8–61.7)	0.004	1 & 2 < 4
Very small TRL-particles, nmol/L	12.5 (1.0–42.5)	26.1 (13.3–51.2)	61.3 (28.5–139.8)	92.4 (45.5–157.3)	<0.001	1 < 3 1 & 2 < 4
Total LDL-particles, nmol/L	1,032 (846–1,230)	1,096 (817–1,418)	1,360 (839–1,708)	1,300 (1,027–1,490)	0.102	All equal
Large LDL-particles, nmol/L	285 (118–363)	172 (53–326)	117 (38–273)	39 (0–347)	0.133	All equal
Medium LDL-particles, nmol/L	424 (184–545)	199 (97–498)	58 (0–392)	0 (0–0)	<0.001	1 > 3 & 4 2 > 4
Small LDL-particles, nmol/L	403 (259–529)	515 (394–836)	682 (506–941)	258 (0–774)	0.005	1 < 3
Total HDL-particles, mmol/L	18.0 (16.1–19.7)	17.2 (15.4–20.5)	13.7 (8.5–17.3)	5.5 (3.7–11.7)	<0.001	1 & 2 > 3 1, 2 & 3 > 4
Large HDL-particles, mmol/L	2.5 (1.5–3.9)	2.7 (1.1–3.6)	0.9 (0.5–1.7)	1.2 (0.7–1.7)	<0.001	1 & 2 > 3 & 4
Medium HDL-particles, mmol/L	3.4 (2.8–3.8)	3.9 (2.8–5.3)	5.1 (2.0–6.4)	3.2 (2.1–5.2)	0.145	All equal
Small HDL-particles, mmol/L	11.9 (9.3–13.4)	11.0 (9.2–12.3)	6.3 (3.0–10.4)	0.7 (0–4.4)	<0.001	1 & 2 > 3 1, 2 & 3 > 4
Lipoprotein X, No. (%)	0 (0%)	1 (3%)	4 (14%)	9 (30%)	<0.001	1 & 2 < 4
Lipoprotein Z, No. (%)	0 (0%)	1 (3%)	5 (18%)	16 (53%)	<0.001	1,2 & 3 < 4
Glyc A, mmol/L	343.3 (314.4–375.8)	397.4 (295.1–512.4)	594.7 (422.2–706.2)	532.2 (442.5–632.3)	<0.001	1 < 3 & 4 2 < 4
Apolipoprotein AI, mmol/L	37.8 (34.7–42.1)	42.5 (34.9–49.4)	33.0 (22.6–40.0)	16.5 (11.9–31.7)	<0.001	1, 2 & 3 > 4
Apolipoprotein B, mg/dl	1.2 (0.9–1.4)	1.2 (0.9–1.5)	1.4 (0.9–2.0)	1.5 (1.3–1.7)	0.008	1 < 4
**HDL cholesterol efflux capacity**	0.80 (0.73–0.97) *N* = 29	0.70 (0.64–0.80) *N* = 28	0.62 (0.55–0.73) *N* = 30	0.55 (0.41–0.70) *N* = 30	<0.001	1 > 2, 3 & 4 2 > 4

IQR, interquartile range; HDL, high density lipoprotein; LDL, low density lipoprotein; TRL, triglyceride rich lipoproteins; mg/dl, milligrams per deciliter; mmol/L, micromoles per liter; MIS-C, multisystem inflammatory syndrome of children; No., number.

* Multiple comparisons. Bonferroni adjusted p-values for 6 comparisons < 0.0083.

**Figure 1:**
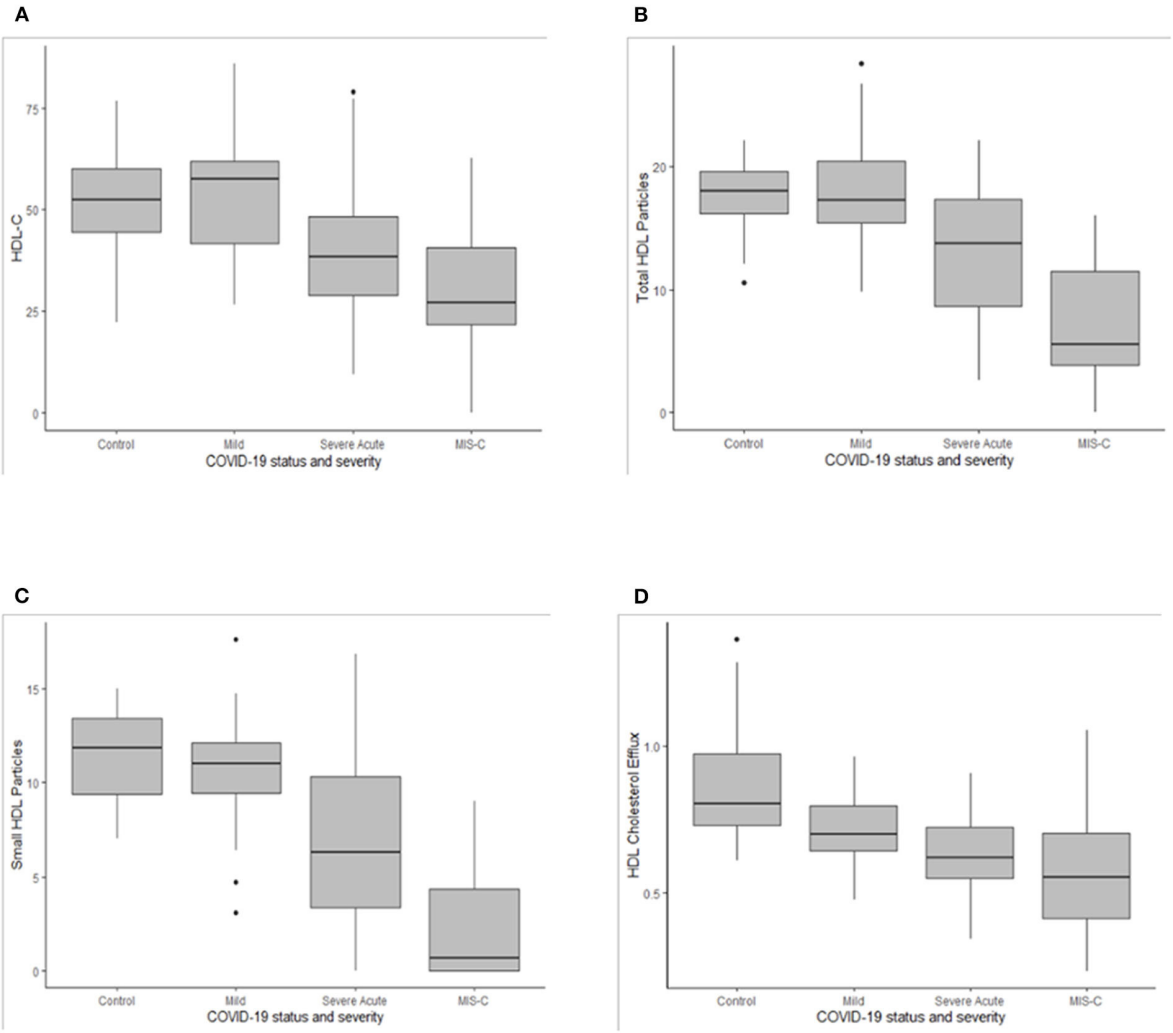
Box plots for high density lipoprotein cholesterol [HDL-c, **(A)**], total HDL particles **(B)**, small HDL particles **(C)**, and the HDL cholesterol efflux **(D)**. Medians are depicted as horizontal black lines, interquartile ranges within the shaded boxes, and ranges as vertical lines for the COVID-19 negative group (Control) and for COVID-19 positive groups with mild or no symptoms (Mild), severe acute symptoms (Severe Acute) or multisystem inflammatory syndrome of children (MIS-C). Asterix depict outliers.

The most striking differences observed were reductions in HDL-c, ApoA-I, total, large and small HDL particles (Table 3 and Figure 1). Controls did not differ from COVID-positive subjects with mild or no symptoms, but HDL-c in both groups was significantly higher than in youth with severe acute symptoms, who in turn had significantly higher HDL-c than those with MIS-C (Figure 1A). The same significant gradient by COVID severity was evident across groupings for ApoA-I, total HDL particles (Figure 1B) and small HDL particles that make the greatest contribution to total particle number (Figure 1C). Large HDL particles did not differ between Controls and COVID-positive youth with mild or no symptoms; both were higher than levels seen with severe acute symptoms or MIS-C. There were no differences across groupings in medium HDL particles.

### Novel biomarkers

GlycA did not differ between Controls and COVID-positive youth with mild or no symptoms but was significantly higher in COVID-positive youth with severe acute symptoms or MIS-C, in comparison to Controls. Levels in MIS-C were also higher than in COVID-positive youth with mild or no symptoms. LP-Z was identified more often in youth with MIS-C. Within the MIS-C group, LP-Z was associated with lower HDL-c, ApoA-I, small HDL particles and HDL CEC (Table 4). The presence of LP-Z was not, however, unique to MIS-C, emerging in a smaller number of COVID-positive youth with severe acute symptoms and one patient with mild or no symptoms.

**Table 4:** LpZ in MISC associates with lower HDL parameters and function.

**Variable, median (IQR)**	**Within MISC group**		
	**LpZ present**	**No LpZ**	***p*-Value**
HDL-c	22.8 (11.5–27.3)	39.4 (27.9–45.0)	0.003
Small HDL particles	0.15 (0–0.75)	4.75 (0.50–6.00)	0.001
ApoA-I	37.8 (19.7–44.0)	89.2 (56.4–104.5)	<0.001
HDL CEC	0.42 (0.33–0.54)	0.68 (0.57–0.75)	0.002

HDL-c, high density lipoprotein-cholesterol; ApoA-I, apolipoprotein A-I; HDL CEC, high density lipoprotein cholesterol efflux capacity.

### HDL cholesterol efflux capacity

High-density lipoprotein CEC among controls was significantly higher than in all three COVID-positive groups, decreasing with increasing COVID-19 severity (Table 3 and Figure 1D). In univariate analyses, demographic factors that demonstrated a significant relationship to HDL CEC included age and race/ethnicity (Table 5). Younger patients had higher CEC; non-Hispanic Black youth had higher CEC, whereas Hispanic youth had lower CEC relative to non-Hispanic White youth. GlycA and the presence LP-Z were inversely associated with HDL CEC in univariate regression. Neither age, race/ethnicity, GlycA, nor LP-Z remained in the multiply adjusted model. Several HDL-associated measures that are highly colinear with ApoA-I, notably HDL particles, total (*r* = 0.944) and small (*r* = 0.726), and HDL-c (*r* = 0.947), all at *p* < 0.001, were associated positively with HDL CEC in univariate analyses. ApoA-I showed the strongest univariate relationship and was included in the multiply adjusted model. ApoA-I and COVID severity together predicted 48% of the variance in HDL CEC.

## Discussion

Neither differential susceptibility nor the predictors of severity of pediatric COVID-19 infections are well understood. The described reduction in HDL-c, ApoA-I, large, small and total HDL-particles, and HDL CEC in direct association with COVID-19 severity in these analyses offers a novel perspective on vulnerability to worse outcomes. Several pre-existing conditions associated with low HDL-c were seen in 85% of COVID-positive youth with severe acute symptoms vs. 39% of those with mild symptoms (*p* < 0.001), recapitulating the differential risk born in this pandemic by adults with pre-existing conditions (1–3). Despite the virtual absence of pre-existing conditions in youth with MIS-C, however, they exhibited the lowest HDL indices. It is not known whether complex genetic factors known to affect HDL function confer differential susceptibility to MIS-C, but adverse consequences of dysfunctional HDL include infection and autoimmune disorders (24). The narrative around HDL has been focused on its antiatherogenic functions but a robust literature also underscores critical roles in innate and adaptive immunity (25–27).

While low HDL-c has previously been linked to worse outcomes in sepsis, specifically respiratory viral sepsis (28), acute infection also actively drives down HDL-c, raising the question of which comes first, low HDL-c or severe infection. Causality in this association is supported by the finding of increased infection in persons with genetically determined low HDL (29). Lipids are not screened universally in pediatrics making it challenging to connect pre-COVID HDL data to these cross-sectional observations, but lipid data in adults before COVID-19 hospitalization identified low HDL-c as a predictor of worse outcomes (30). Both preexisting HDL deficiencies and consumption of HDL in the face of infection likely pertain; limited HDL reserves may be depleted faster. It has previously been observed that small HDL particles, the HDL subspecies most severely diminished in this study as well as in severe adult COVID-19 (21), is also the HDL subspecies most strongly associated with CEC function (31).

A series of observations links HDL function to immunity *via* at least two pathways. The first relates to a bioactive lipid, sphingosine 1 phosphate (S1P), that in circulation, preferentially resides on HDL and acts through S1P receptors to regulate immunomodulatory transcriptional pathways (32). A dedicated HDL apolipoprotein, ApoM, is the preferred chaperone for S1P. HDL containing apo-M stimulate cholesterol efflux more efficiently than HDL without ApoM (33, 34). ApoM-S1P also plays a role in lymphocyte trafficking, restraining lymphopoiesis by activating S1P receptors on bone marrow lymphocyte progenitors (35). The cytokine storm seen in influenza and H1N1 viral sepsis can be attenuated by S1P modulation with S1P receptor agonist therapy (36). Dysfunctional HDL lacking this ApoM-S1P immunomodulatory role may help explain the hyperactivated T cell responsiveness described in youth with MIS-C (37). HDL ApoM-S1P has not been measured directly in pediatric COVID-19, but low circulating apoM (20) and S1P (38) in adult COVID-19 predict both disease severity and mortality.

A second potential mechanistic pathway invokes a role for HDL CEC in innate immunity, via neutrophil extracellular traps (NETs), web-like networks of decondensed chromatin, histones, and neutrophil proteins that ensnare novel microbial bacterial, parasitic, and viral pathogens before an antibody response can be mounted (39). Normally, the process of NET formation, or NETosis, is self-contained once the offending agent is neutralized. Dysregulated, excessive neutrophil infiltration and NETosis have been found in many chronic inflammatory conditions (40). Traditionally HDL CEC research has been focused on clearance of macrophage foam cell cholesterol within atherosclerotic plaques (41, 42). The link between functional HDL CEC and NETosis was first proposed by Westerterp et al. (43) with the observation that excessive cholesterol within myeloid cells activates the NLR Family Pyrin Domain Containing three inflammasome known to both enhance white blood cell accumulation and destabilize NETs (43–45). Inflammasome activation and dysregulated NETosis have specifically been identified as predictors of severity in adult COVID-19 (46, 47). A recent preprint also documented signatures of neutrophil activation and degranulation with high levels of spontaneous NET formation in fresh whole blood of pediatric patients with MIS-C (48). Interestingly, enhanced NETosis has been implicated in Kawasaki disease (KD) (49), as has disordered lipid metabolism, specifically low HDL-c and HDL particles (50). Furthermore, lower apoAI levels were recently associated with resistance to intravenous immunoglobulin therapy and worse coronary artery lesions in a prospective cohort study of KD (51). KD, beyond clinical parallels with MIS-C, has long been suggested to occur following an elusive infectious trigger. No single pathogen has been identified, but the possibility exists that KD and MIS-C both represent dysregulated immunity triggered by infection that occurs in a host naïve to novel pathogens (52). Susceptibility to immune dysregulation may relate to functional HDL capacity in the host.

**Table 5:** Modeling of high density lipoprotein cholesterol efflux capacity.

	**Univariate**		**Multivariate**	
			**(*R*^2^ = 0.484)**	
	**Estimate**	***p*-Value**	**Estimate**	***p*-Value**
**ApoA1**	**0.0032**	**<0.001**	**0.0029**	**<0.001**
Glyc A	−0.0004	<0.001	−0.0014	0.137
Age	−0.0037	0.180	0.0005	0.819
**COHORT**				
Control	–	**<0.001**	–	**0.018**
Mild or No Sx	−0.1392		−0.1368	
Severe acute Sx	−0.2144		−0.0858	
MIS-C	−0.2762		−0.1052	
**Race/Ethnicity**				
Caucasian	–	**0.042**	–	0.981
Black	0.0734		−0.0125	
Hispanic	−0.0428		−0.0084	
Unknown	0.0029		−0.0312	
**Presence LpZ**	−0.2172	**<0.001**	0.0042	0.936

The variables listed were analyzed in both univariate and multivariate regression and the estimates and *p*-values listed. Significant *p*-values are bolded. In the final multiply adjusted model, ApoA-I and the Cohort (grouped by symptom severity) explain 48% of the variance in HDL cholesterol effux capacity.

ApoA-I, apolipoprotein A-I; HDL, high density lipoprotein; Sx, symptoms; LpZ, lipoprotein Z, MIS-C, multisystem inflammatory syndrome of children.

A subtext to these findings is rooted in the evidence that therapeutic lifestyle change improves HDL function (53). Limited pharmacologic options also exist, notably fibric acid derivatives that work *via* global transcriptional pathways also activated by polyphenolic and omega 3 fatty acid nutraceuticals (54) present in the foods most often missing from the American diet (55, 56), particularly among persons at socioeconomic disadvantage (57). Recombinant HDL is being explored as a new COVID-19 treatment (58), but exercise (59) and a balanced, fiber-rich, plant-centered diet (60) are proven, if underutilized strategies known to improve HDL function. Retrospective analysis of 2,884 health care workers in six countries severely impacted by the COVID-19 pandemic (61) and of 343,850 adults followed in the UK Biobank (62), as well as a prospective smartphone-based COVID symptom study of 592,571 UK and US participants (63) all associated significantly less symptom severity with healthier lifestyle practices. The findings reported upon here suggest one mechanism, improved HDL function, that may mediate the observed association between therapeutic lifestyle change and strengthened immunity against COVID-19. Both psychological and lifestyle factors that optimize HDL function are also known to optimize the immune response to vaccination (64).

Other biomarkers previously associated with COVID severity, namely CRP (7), RDW (65), and GlycA (21), were also associated with more severe outcomes. LP-Z, a triglyceride and free cholesterol-enriched, cholesteryl ester-depleted LDL particle not normally present, was identified in 53% of COVID positive youth with MIS-C, 18% with severe acute symptoms and 3% with mild or no symptoms. The presence of LP-Z may reflect the inflammatory hepatitis and cholestasis described in both adult COVID-19 (66, 67) and up to one third of SARS CoV-2 infections in youth (68). LP-Z in adults has specifically been linked to worse outcomes in alcoholic cirrhotic disease that also associates with poor HDL function (69). This is the first description of LP-Z in youth and the increased occurrence not only among youth with MIS-C, but among those with the lowest HDL parameters, is striking.

These analyses are limited by their purely observational nature and the lack of data on study participants either preceding or following their presentation with or without COVID-19. Despite national recommendations, lipid screening is still only done on a minority of children and adolescents in the US (70). COVID-negative controls in our analyses were younger and a larger proportion were non-Hispanic Black than in the COVID-positive groups, reflecting the population served in our inner-city outpatient programs. Younger age may relate to the slightly higher HDL CEC among COVID-negative controls as compared with mild or asymptomatic COVID-positive youth in univariate analyses, but neither age nor race/ethnicity remained in the multiply adjusted model for HDL CEC. Controls did not differ from COVID positive youth with mild or no symptoms in any other HDL parameters. Incomplete clinical lab data could be extracted but complete NMR lipoprotein analyses and HDL CEC were performed from which the main findings in this study emerged. Future studies will be needed to explore the possible mechanism(s) behind our findings, such as the measurement of apo M and S1P content in HDL and measures of inflammasome activation and NETosis together with HDL functional assays in both acute and long COVID-19.

In summary, as has been appreciated in adults, lower HDL-c, large and small HDL particles, and HDL CEC are associated with COVID-19 severity in youth, diminishing sequentially across the range of increasing symptoms. The atypical lipoprotein, LP-Z, emerges among the youth with lowest HDL function. The functional properties of HDL, enhanced by heart-healthy physical activity and nutrition, represent a largely untapped therapy to improve immune resilience to SARS CoV2 and potential future novel pathogens. The association of diminished HDL parameters in MIS-C may help inform our understanding of Kawasaki disease. Increased prospective screening of lipids, including phenotyping available at reference labs, may help further elucidate the role played by HDL in immunity.

## Data availability statement

The original contributions presented in the study are included in the article/Supplementary material, further inquiries can be directed to the corresponding author.

## Ethics statement

The studies involving human participants were reviewed and approved by Children's National Hospital Institutional Review Board. Written informed consent for participation was not provided by the participants' legal guardians/next of kin because: only de-identified reserve plasma that would normally have been discarded, was saved and linked to the patient's COVID-19 status to permit these analyses.

## Author contributions

MM-S, AR, and NM conceptualized and designed the study. WS contributed to the acquisition of data by identifying frozen plasma aliquots for the study cohort and extracting associated clinical data from charts. MD designed and executed the IRB protocol authorizing creation of a registry of reserve plasma from pediatric patients screened for COVID-19 that permit studies like the current analyses. RD is a member of the Children's National MIS-C Taskforce (Director) and COVID Taskforce and assisted with the identification and categorization of study subjects. MP contributed to the acquisition of data by conducting the HDL functional cholesterol efflux assays. MP and RB contributed to the acquisition of data by conducting NMR LipoProfile^®^ analyses. JB performed all statistical analyses, assisted with the preparation of all tables and figures. JO provided consultation on the interpretation of the NMR LipoProfile^®^ data. MD, NM, and AR assisted with funding. MM-S drafted the initial manuscript and incorporated all edits from the authorship team. All authors reviewed and approved of the final manuscript as submitted and agree to be accountable for all aspects of the work.

## Funding

This study was supported by Award Number UL1TR001876 from the NIH National Center for Advancing Translational Sciences. Research by Playford, Ballout, Mehta, and Remaley was supported by DIR intramural research funds from the National Heart, Lung and Blood Institute. The other authors received no additional funding.

## Conflict of interest

Author JO is a consultant, stockholder, and former employee of Labcorp, the commercial provider of the NMR LipoProfile testing system used at the NIH. The remaining authors declare that the research was conducted in the absence of any commercial or financial relationships that could be construed as a potential conflict of interest.

## Publisher's note

All claims expressed in this article are solely those of the authors and do not necessarily represent those of their affiliated organizations, or those of the publisher, the editors and the reviewers. Any product that may be evaluated in this article, or claim that may be made by its manufacturer, is not guaranteed or endorsed by the publisher.

## Author disclaimer

The contents are solely the responsibility of the authors and do not necessarily represent the official views of the National Center for Advancing Translational Sciences or the National Institutes of Health.
